# Combination of ultra-purified stem cells with an *in situ*-forming bioresorbable gel enhances intervertebral disc regeneration

**DOI:** 10.1016/j.ebiom.2022.103845

**Published:** 2022-01-25

**Authors:** Daisuke Ukeba, Katsuhisa Yamada, Takashi Suyama, Darren R. Lebl, Takeru Tsujimoto, Takayuki Nonoyama, Hirokazu Sugino, Norimasa Iwasaki, Masatoki Watanabe, Yumi Matsuzaki, Hideki Sudo

**Affiliations:** a Department of Orthopedic Surgery, Faculty of Medicine and Graduate School of Medicine, Hokkaido University, Sapporo, Hokkaido, Japan; b PuREC/Bio-venture from Shimane University, Izumo, Shimane, Japan; c Department of Orthopedic Surgery, Spine Service, Hospital for Special Surgery, New York, USA; d Global Institution for Collaborative Research and Education, Global Station for Soft Matter, Hokkaido University, Sapporo, Hokkaido, Japan; e Department of Cancer Pathology, Faculty of Medicine and Graduate School of Medicine, Hokkaido University, Sapporo, Hokkaido, Japan; f Japan Tissue Engineering Co., Ltd. (J-TEC), Gamagori, Aichi, Japan; g Department of Advanced Medicine for Spine and Spinal Cord Disorders, Faculty of Medicine and Graduate School of Medicine, Hokkaido University, N15W7, Sapporo, Hokkaido 060-8638, Japan

**Keywords:** Lumbar intervertebral disc herniation, Combined lumbar canal stenosis, Ultra-purified clonogenic bone marrow-derived mesenchymal stem cell, *In situ*-forming gel, Intervertebral disc regeneration

## Abstract

**Background:**

Lumbar intervertebral disc (IVD) herniations are associated with significant disability. Discectomy is the conventional treatment option for IVD herniations but causes a defect in the IVD, which has low self-repair ability, thereby representing a risk of further IVD degeneration. An acellular, bioresorbable, and good manufacturing practice (GMP)-compliant *in situ*-forming gel, which corrects discectomy-associated IVD defects and prevents further IVD degeneration had been developed. However, this acellular matrix-based strategy has certain limitations, particularly in elderly patients, whose tissues have low self-repair ability. The aim of this study was to investigate the therapeutic efficacy of using a combination of newly-developed, ultra-purified, GMP-compliant, human bone marrow mesenchymal stem cells (rapidly expanding clones; RECs) and the gel for IVD regeneration after discectomy in a sheep model of severe IVD degeneration.

**Methods:**

RECs and nucleus pulposus cells (NPCs) were co-cultured in the gel. In addition, RECs combined with the gel were implanted into IVDs following discectomy in sheep with degenerated IVDs.

**Findings:**

Gene expression of NPC markers, growth factors, and extracellular matrix increased significantly in the co-culture compared to that in each mono-culture. The REC and gel combination enhanced IVD regeneration after discectomy (up to 24 weeks) in the severe IVD degeneration sheep model.

**Interpretation:**

These findings demonstrate the translational potential of the combination of RECs with an *in situ*-forming gel for the treatment of herniations in degenerative human IVDs.

**Funding:**

Ministry of Education, Culture, Sports, Science, and Technology of Japan, Japan Agency for Medical Research and Development, and the Mochida Pharmaceutical Co., Ltd.

**Research in context**

**Evidence before this study**

Discectomy is the conventional surgical procedure involving the removal of intervertebral disc (IVD) materials compressing the nerve root. However, discectomy causes a defect in IVDs, which have low self-repair ability, posing a substantial risk of further degeneration of the IVD. Previously, we demonstrated that combination of allogenic bone-derived mesenchymal stem cells (BMSCs) and an *in-situ* forming gel was effective at inducing IVD regeneration. However, BMSCs used for clinical studies often lead to variable or even contradictory findings because these BMSCs always include non-differentiating contaminant cells. To overcome these limitations, we developed genetically stable, ultra-purified, clonogenic BMSCs (known as rapidly expanding clones; RECs) that exhibited all the functions of BMSCs and did not show lot-related differences in clinical applications.

**Added value of this study**

The present study demonstrates that the expression levels of nucleus pulposus cell (NPC) markers, growth factors, and extracellular matrix components were significantly increased in the co-culture of human NPCs and RECs compared with those observed in the co-culture of NPCs with commercially available BMSCs. The efficacy of the combination of RECs and the *in-situ* forming gel was observed at the site of IVD degeneration in a sheep lumbar spine model.

**Implications of all the available evidence**

The findings reveal that the combination of ultra-purified BMSCs in the form of RECs and *in situ*-forming gel enhances IVD regeneration after discectomy in a sheep model exhibiting severe IVD degeneration, without resulting in neoplastic changes, and enable the maintenance of mechanical functions after implantation. These findings and strategy need to be validated in clinical settings in future studies, especially in an elderly cohort such as individuals with combined lumbar canal stenosis.

Alt-text: Unlabelled box

## Introduction

Lumbar intervertebral disc (IVD) herniations, which can cause sciatica, are associated with significant disability and a decreased quality of life.^1^ Discectomy is the conventional surgical procedure involving the removal of IVD materials compressing the nerve root to relieve symptoms of IVD herniations.^1^, ^2^, ^3^, ^4^ However, discectomy causes a large defect in IVDs, which have low self-repair ability, posing a substantial risk of further progression and degeneration of the IVD.^2^, ^3^, ^4^ The prevalence of re-herniation has been reported to be between 5 and 26% of approximately 500,000 lumbar discectomies per year.^4^, ^5^, ^6^ Consequently, patients are likely to experience disabling discogenic lower back pain as well as reherniation that may require additional surgery, such as repeated discectomy or intervertebral fusion.^5^^,^^7^

Integration of a next-generation surgical procedure that could prevent further IVD degeneration following the discectomy procedure might improve long-term surgical outcomes. Recent studies have demonstrated that cell transplantation and biomaterials could potentially be used to treat IVD degeneration.^2^, ^3^, ^4^^,^^8^, ^9^, ^10^, ^11^, ^12^, ^13^, ^14^, ^15^, ^16^ The transplantation of bone marrow-derived mesenchymal stem cells (BMSCs) into IVDs has shown promise for the treatment of chronic lower back pain.^10^ However, BMSC transplantation has several limitations. Traditionally, the isolation of BMSCs from the unfractionated, whole bone marrow has been retrospectively confirmed based on their adherence to plastic dishes as fibroblast-like colony-forming cells.^17^^,^^18^ Consequently, it has been difficult to maintain cell quality, because the cell population harvested using this method always includes non-differentiating contaminant cells.^18^^,^^19^ Extended cell culture is often needed to remove contaminant cells and isolate a moderately pure BMSC population.^18^ Moreover, the proliferation, differentiation, and migration abilities of BMSCs gradually decrease as the cells start exhibiting a more mature phenotype during culture.^20^^,^^21^ Consequently, BMSCs used for clinical studies often lead to variable or even contradictory findings.^22^^,^^23^

**Figure 1:**
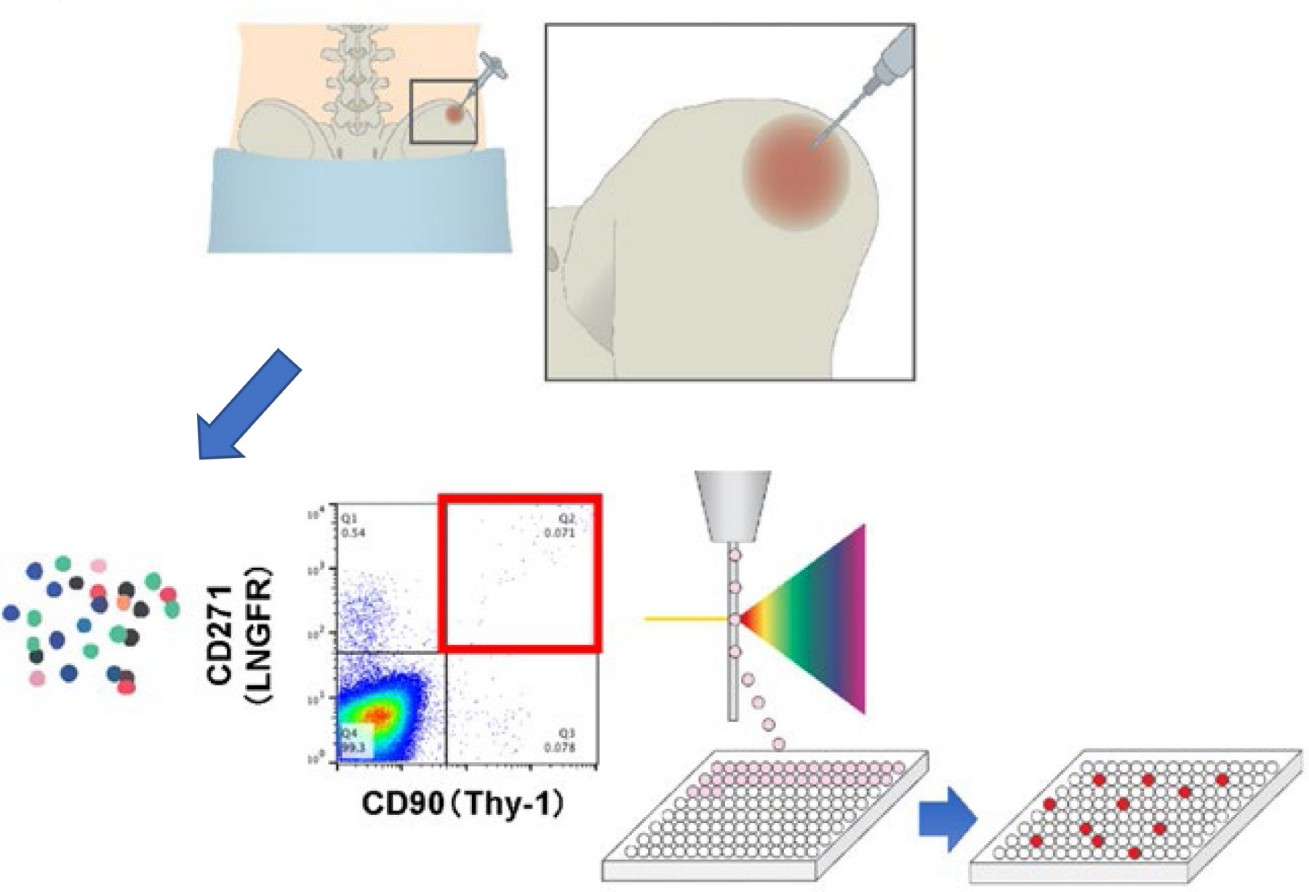
Schematic diagram demonstrating REC isolation. Flow cytometric profiles of human bone marrow cells stained for CD 271 (LNGFR) and CD 90 (THY-1). Cells collected from 1 well are seeded in a 35mm culture dish and expanded up to 14 days to obtain a uniform cell population with high differentiation and proliferation ability.

To overcome these limitations, we developed a single cell isolation technique for directly obtaining BMSCs from the bone marrow by sorting cells based on the expression of surface markers.^18^^,^^24^, ^25^, ^26^, ^27^ Human BMSCs were isolated from the bone marrow based on the expression of two cell-surface markers (CD271 and CD90) as according to the findings of previous studies, the use of these two markers serves as the best combination for the efficient collection of BMSCs.^18^^,^^26^^,^^27^ After cell isolation and expansion, we obtained genetically stable, ultra-purified, good manufacturing practice (GMP)-compliant, clonogenic BMSCs (known as rapidly expanding clones; RECs) that exhibited all the functions of BMSCs and did not show lot-related differences in clinical applications (Figure 1).^18^

A carrier biomaterial is necessary to prevent cell leakage during discectomy.^2^^,^^15^ To date, no biomaterials have been clinically approved for this purpose because each biomaterial did not meet criteria for both biologic and mechanical safety. We had previously developed an original, acellular, bioresorbable, ultra-purified alginate (UPAL) gel to repair IVD degeneration in small^2^^,^^3^^,^^15^^,^^16^ and large animal models *in vivo*.^3^ Alginate is a polysaccharide derived from brown seaweed, *Phaeophyceae*, and exhibits low-level endotoxicity (1/10,000^th^ that of commercially available laboratory alginate), and thus precludes immunologic reactions.^3^ This material gelates within 5 min *in situ* via calcium ion-mediated crosslinking and exhibits appropriate biomechanical characteristics, without resulting in material protrusion after discectomy.^3^^,^^15^ In addition, the UPAL gel showed a lack of immunogenicity during *in vivo* biological safety testing, performed according to International Organization for Standardization and Good Laboratory Practice (GLP) standards.^3^ Consequently, we are currently conducting a first-in-human clinical trial using an acellular matrix-based strategy and implanting this GMP-compliant gel into the defect after discectomy in young patients (age, 20–40 y).^28^

We have previously demonstrated that the intrinsic nucleus pulposus (NP) and NP progenitor cells transmigrate into the UPAL matrix for endogenous IVD repair.^3^ Because residual IVD tissues observed after discectomy act as a possible source of reparative cells and the grade of IVD degeneration affects repair capabilities, the strategy of using UPAL gel alone may have limitations in an elderly population, as it could result in the development of conditions such as combined lumbar canal stenosis (e.g., IVD herniation complicated by lumbar canal stenosis).^2^^,^^3^ Notably, most animal models of IVD degeneration were created using healthy IVDs and therefore might exhibit natural healing capabilities.^3^^,^^4^^,^^8^^,^^11^, ^12^, ^13^, ^14^^,^^16^ To identify the course of post-surgical IVD degeneration from the onset, we generated a rabbit model of IVD degeneration.^2^^,^^15^ We demonstrated that following discectomy, the use of UPAL gel alone suppressed IVD degeneration compared to the extent of degeneration without UPAL gel treatment. Additionally, the combination of allogenic BMSCs and UPAL gel was more effective at inducing IVD regeneration compared to UPAL alone.^2^^,^^15^

To further advance the clinical translation to enhance IVD regeneration post-discectomy for degenerated IVDs, we developed a large animal ovine model of IVD degeneration and successfully implanted RECs encapsulated in the UPAL gel into the IVDs—as a medical product—in a GLP-adapted laboratory. We hypothesized that the implantation of RECs encapsulated in the UPAL gel would promote spontaneous IVD regeneration after discectomy. Here, we aimed to demonstrate that the combination of these GMP-compliant, ultra-purified, BMSCs and *in situ*-forming gel enhances IVD regeneration after discectomy in a sheep model exhibiting severe IVD degeneration, without resulting in neoplastic changes, and enable the maintenance of mechanical functions after implantation.

## Methods

### Study design

The primarily explored parameters in this study were: (i) potential mechanisms of IVD regeneration via the 3-dimensional (3D) co-culture of human NPCs and RECs *in vitro*; (ii) comparison of the effects of RECs and commercial human BMSCs on NPCs via the 3D co-culture of these cells with NPCs; (iii) measurement of the mechanical properties of UPAL and REC + UPAL gels; (iv) evaluation of regenerative capacity of severely degenerated IVDs after discectomy *in vivo*; and (v) evaluation of tumorigenesis in the implanted IVDs.

Healthy human NPCs and RECs were co-cultured in a 3D system in UPAL gel to evaluate the mechanism underlying IVD regeneration. At 0 and 7 days after culture, we analyzed the expression of NPC markers, including HIF-1α, GLUT-1, and brachyury; growth factors, including CDMP-1, TGF-β, and IGF-1; and extracellular matrix (ECM) components, including Type II collagen and aggrecan (Table 1), in all cell types, using quantitative reverse transcription polymerase chain reaction (qRT-PCR) (n = 4).^2^^,^^9^^,^^29^^,^^30^ In addition, NPCs and commercial BMSCs were co-cultured to compare the effects of RECs and commercial BMSCs on NPCs. Then, we measured the mechanical properties of UPAL and REC + UPAL gels using both unconfined and confined compression tests (see *2.12. Elasticity analysis of the gels using both unconfined and confined compression tests*). Disc-shaped gels were compressed at a constant speed of 0·5 mm/min, and Young's moduli were calculated (n = 4).^3^^,^^15^^,^^31^^,^^32^

**Table 1:** Overview of the targeted genes.

Main role	Gene product
NPC makers	HIF-1α
	GLUT-1
	Brachyury
Growth factors	CDMP-1
	TGF-β
	IGF-1
Extracellular matrix (ECM) components	Type II collagen
	Aggrecan

In animal procedures, 14 sheep (56 IVDs in total) were divided into the following four groups: Intact control (4 weeks, n = 6; 24 weeks, n = 6), Discectomy (4 weeks, n = 6; 24 weeks, n = 6), Gel (4 weeks, n = 8; 24 weeks, n = 8), and REC + gel (4 weeks, n = 8; 24 weeks, n = 8) groups. In a preliminary study, we found that IVD degeneration occurred after 4 weeks, with the removal of 20 mg or more of NP tissue in a sheep model (Supplementary Figure 1). Consequently, to create severely degenerated IVDs, we initially removed 20 mg of NP tissue from the treated IVDs and further removed 70 mg of the gel-like NP tissue separate from the AF from the degenerated IVDs, four weeks after the initial surgery. For the second removal procedure, the amount of 70 mg was determined based on the ratio of the removed amounts of normal and degenerated IVDs in previous studies.^2^^,^^3^ In a previous study, the total amount of NP tissue in a rabbit normal IVD was 15-18 mg, whereas the amount in a rabbit degenerated IVD, created by needle puncture was 10-12 mg, corresponding to approximately 70% of the amount in the normal IVD.^2^ Since the amount of NP tissue removed from a sheep normal IVD was 100 mg in our previous study,^3^ the amount of NP tissue removed from a degenerated IVD in this study was set to 70 mg, which was equivalent to 70% of the amount of NP tissue in the normal IVD. After the second discectomy, UPAL or a solution containing a combination of RECs with UPAL was implanted into the IVD cavity. Sheep were euthanized 4 and 24 weeks after implantation. IVDs were qualitatively analyzed to assess IVD degeneration using a 3·0-T magnetic resonance imaging (MRI).^2^^,^^3^^,^^15^^,^^33^^,^^34^ Subsequently, IVDs were stained with hematoxylin & eosin (H&E) and safranin-O for histological analysis, and immunohistochemistry (IHC) was performed to evaluate the levels of Type II and I collagen for analyzing the ECM components. Finally, tumorigenesis analysis was performed using histological specimens.^2^^,^^3^^,^^15^

### Ethics statement

The ethics committee of the Hokkaido University Graduate School of Medicine approved the use of healthy human IVDs. Animal procedures were approved by the Institutional Animal Care and Use Committee at Hokkaido University and Hamri Co., Ltd. (Ibaraki, Japan), and were performed according to the guidelines recommended by these committees.

### Preparation of RECs

Frozen GMP-compliant RECs provided by Japan Tissue Engineering, Inc. were used in this study. In the comparative analysis of RECs and commercial human BMSCs, we prepared three clones of RECs, namely, REC-02 prototype #003-P6-191220, REC-02 HLWF-1-P6-210114, and REC-02 QRKF-1-P6-210210. They were prepared by the company and received in passage 6. The cells were thawed in a warm bath at 37ºC prior to 3D culture and were directly used after thawing.

### Preparation of commercial human BMSCs

Commercially available human BMSCs were obtained (hMSC-BM; PromoCell, Heidelberg, Germany; C-12974, lot number: 412Z022.4) as described previously.^2^ BMSCs were cultured with a complete culture medium, namely, Dulbecco's modified Eagle's medium (with low glucose levels; 2 mg/mL) containing L-glutamine and phenol red (DMEM; FUJIFILM Wako Pure Chemical Corporation, Osaka, Japan), supplemented with 20% HyClone fetal bovine serum (FBS; Cytiva, Tokyo, Japan), 1% penicillin/streptomycin, 1·25 mg/mL fungizone (Life Technologies, Thermo Fisher Scientific, Waltham, MA, USA), 1% HEPES (Life Technologies, Thermo Fisher Scientific), and 0·1% bFGF (Kaken Pharmaceutical Co., Ltd., Tokyo, Japan) in a humidified atmosphere (20% O_2,_ 5% CO_2_, 37°C) according to the manufacturer's instructions. The medium was replaced twice per week, and BMSCs from the fourth passage process were used (six passages in total similar to that with RECs).

### Preparation of UPAL gel and 3D culture

The UPAL gel (Mochida Pharmaceutical Co. Ltd., Tokyo, Japan) was used as an alginate scaffold for 3D culture, as described previously.^2^^,^^3^ We prepared a 2% (w/v) UPAL solution dissolved in phosphate-buffered saline (PBS; FUJIFILM Wako Pure Chemical Industries) and used CaCl_2_ solution (102 mM) for gelation. RECs or commercial BMSCs were mixed with the UPAL solution at a final cell concentration of 1 × 10^6^ cells/mL.^2^^,^^30^^,^^35^ The cell-UPAL mixed solution, total 1 mL in each group, was pipetted into a 102 mM CaCl_2_ solution (100mL) using a 22-gauge needle for gelation. The two types of the gel obtained in the shape of beads were cultured with the medium for 7 days in a humidified atmosphere (20% O_2,_ 5% CO_2_, 37°C). The medium was changed every 3 days. To retrieve cells after 7 days of 3D culture, we collected the cells from gel beads after dissolution using 55 mM sodium citrate and centrifugation (110 × *g* for 10 min at 4°C), as described previously.^2^^,^^3^ Additionally, we used the RECs and commercial BMSCs prepared as plane cultures (2D culture) for 7 days under normal condition as the control group, as mentioned previously (i.e., the following four groups were set up as the experimental groups: 1) 2D-cultured RECs, 2) 3D-cultured RECs, 3) 2D-cultured BMSCs, and 4) 3D-cultured BMSCs).

### Analysis of cell proliferating capacity

After 7 days of 2D or 3D culture, cells obtained in the 4 experimental groups (see *2.5. Preparation of UPAL gel and 3D culture*) were seeded again as a secondary culture. We collected the cells at confluency and evaluated their cell proliferative capacity based on the doubling time (Td) defined as the time required for the cell number to double. Td was calculated using the following formula:

Td = (t_2_ − t_1_) × ln (2) / ln (N_2_ / N_1_)

where N_2_ and N_1_ were the cell number at time t_2_ and t_1_.

### Flow cytometric analysis

PBS (with 2% of FBS) was added to each of the four types of cells to prepare cell suspensions at a final density of 1 × 10^6^ cells/mL. Then, the cells were stained anti-CD90 antibodies (PE anti-human CD90 (Thy1), BioLegend, San Diego, CA, USA; Cat# 328109, RRID: AB_893442) and anti-CD45 antibodies (FITC anti-human CD45, Beckman Coulter, Brea, CA, USA; Cat# IM0782U, RRID: AB_131000). We also performed staining with mouse IgG1 antibodies (Mouse IgG1-PE, BioLegend; Mouse IgG1-FITC, Beckman Coulter) as negative controls. Propidium iodide (PI) was used to detect dead cells. Flow cytometric analysis was performed using CytoFLEX System (Beckman Coulter) to evaluate cell viability and the uniformity and positivity of each cell surface antigen. FlowJo software (Becton Dickinson, Franklin Lakes, NJ, USA) was used for data analysis.

### Preparation of healthy human NPCs

Human NP samples were obtained from nine patients (mean age ± standard deviation (SD), 15·3 ± 3·3 years) who had undergone anterior spinal fusion for adolescent idiopathic scoliosis at Hokkaido University Hospital. We obtained written informed consent from all participants of this study. The samples were obtained during the surgical procedure. All IVDs were analyzed before surgery via MRI and graded for degenerative changes using the Pfirrmann classification system.^36^ MRI measurement was performed within 3 months before surgical removal. All IVDs were classified as grade 1 IVDs, suggesting that all the samples were non-degenerated IVDs.

NP cells were isolated within 1 hr after the harvesting of human IVDs during the surgery and cultured as described previously.^2^^,^^3^ Briefly, each gel-like NP was separated from the annulus fibrosus (AF) under a dissecting microscope. The tissue specimens were placed in the medium as described above (section 2.4). The preparations were washed twice via centrifugation (200 × *g*, 3 min) and resuspended in the medium supplemented with 0·25% collagenase. For cell isolation, the preparations were incubated in a shaking incubator (37°C, 4 h) and then centrifuged twice (200 × *g*, 3 min). Cells that were separated from the matrix were placed in 10 cm tissue culture dishes and incubated under the conditions indicated in section 2.4. The medium was changed twice a week, and NPCs from the fourth passage were used.

### Preparation of RECs and commercial human BMSCs

The RECs (REC-02_prototype #003-P6-191220) and commercial human BMSCs as above were also used for 3D co-culture. Two types of the cells were cultured according to the manufacturer's instructions in the same way as the 2D culture above. The medium was replaced twice a week. Both of cells from the second passage (RECs, 8 passages in total; BMSCs, 4 passages in total) were used.

### 3D co-culture and mono-culture

We prepared a 2% UPAL solution and used CaCl_2_ solution (102 mM) for gelation, as described previously.^2^^,^^3^ In advance of the 3D culture, the RECs and BMSCs were labeled with 20 mM 5,6-caboxyfluorescein diactetate succinimidyl ester (CFDA-SE) (CFDA-SE Cell Proliferation Assay Kit; BIO RAD, Hercules, CA, USA) fluorescently, referring to the manufacturer's manuals.^2^^,^^15^^,^^37^ Afterward, the labeled cells (BMSCs or RECs) and unlabeled NPCs were mixed (i.e. a combination of two cell types, NPCs + BMSCs or NPCs + RECs) with the UPAL solution at the same ratio (each cell 1 × 10^6^ cells/mL respectively),^2^^,^^30^^,^^35^ with a final cell concentration of 2 × 10^6^ cells/mL. The cell-UPAL mixed solution was pipetted into CaCl_2_ solution of 102 mM using a 22-gauge needle to gelate. The obtained gels were cultured with the medium for 7 days under hypoxic conditions (5% O_2_ and 5% CO_2_).^2^^,^^35^ Additionally, NPCs or RECs were mixed in the UPAL solution separately at a concentration of 1 × 10^6^ cells/mL. The cell concentration was selected on the basis of the results of a preceding research.^2^ After gelation, the gel beads were cultured in the same manner, under hypoxic conditions.

**Figure 2:**
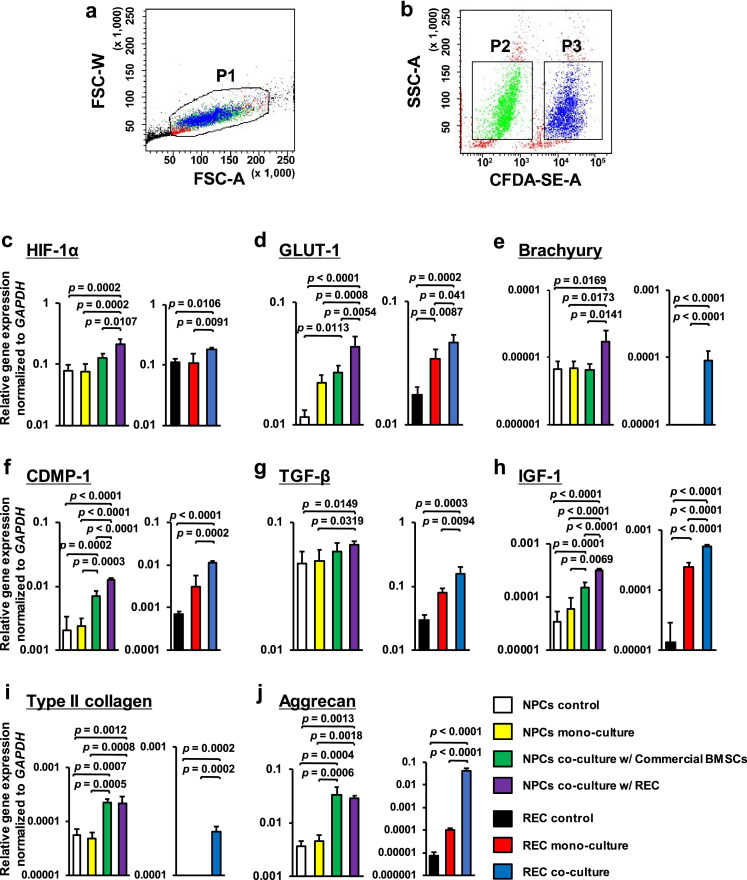
Expression profiles of human nucleus pulposus cells (NPCs) and rapidly expanding clones (RECs) after 7 days of 3-dimensional (3D) co-culture. **(a,b)**: Cell sorting data are used to distinguish between 5,6-caboxyfluorescein diactetate succinimidyl ester (CFDA-SE)-labeled RECs and unlabeled NPCs. **(a)**: The P1 gate excludes dead cells and debris live cells. **(b)**: A 2D dot plot showing unlabeled NPCs at the P2 gate and CFDA-SE-labeled RECs at the P3 gate. **(c-j)**: Gene expression levels for each cell were normalized to those of the housekeeping gene *GAPDH* and plotted on a log scale (y-axis). Data obtained from four different human NPC lines were averaged. **(c)**: HIF-1α, **(d)**: GLUT-1, **(e)**: brachyury, **(f)**: CDMP-1, **(g)**: TGF-β, **(h)**: IGF-1, **(i)**: Type II collagen, and **(j)**: aggrecan. Data are shown as means ± SD values (n = 4). The expression of the genes encoding brachyury and type II collagen was not detected in both the REC control and REC mono-culture groups. Significant differences were assessed by one-way ANOVA with a post hoc Tukey-Kramer test. FSC-A, forward scatter-area; FSC-W, forward scatter-width; SSC-A, side scatter-area; CFDA-SE-A, CFDA-SE-area.

At 0 and 7 days after culture, all gel beads were dissolved in 55 mM sodium citrate until the gel was separated from the cells as above. In the co-cultured groups, collected cells were sorted using a BD FACSAria III High speed cell sorter using Diva software version 7·0 (BD Biosciences, San Jose, CA, USA).^2^^,^^30^ Fluorescent cell with 530 nm were selected as RECs and BMSCs, and non-fluorescing cells were selected as NPCs after the excluding debris and dead cells (Figure 2a and b). Subsequently, we had 7 experimental groups (Table 2).

**Table 2:** Experimental groups.

Cell type and culture condition	Experimental Group
Non-cultured NPCs (day 0)	NPCs control
Mono-cultured NPCs (day 7)	NPCs mono-culture
NPCs co-cultured with commercial BMSCs (day7)	NPCs co-culture w/ Commercial BMSCs
NPCs co-cultured with REC (day 7)	NPCs co-culture w/ REC
Non-cultured RECs (day 0)	REC control
Mono-cultured RECs (day 7)	REC mono-culture
RECs co-cultured with NPCs (day 7)	REC co-culture

### RNA extraction and qRT-PCR

The collected cells were lysed in 1 mL of TRIzol® (Invitrogen, Thermo Fisher Scientific), and total RNA was extracted from the samples using the RNeasy Mini kit (Qiagen, Valencia, CA, USA). Real-time qRT-PCR was performed using TaqMan® Gene Expression Assays (Applied Biosystems, Thermo Fisher Scientific). Assay IDs used for the assay are presented in Supplementary Table 1. We obtained a cycle threshold (Ct) value for each sample. Moreover, the relative mRNA expression levels of each target gene, NPC markers, growth factors, and ECM components were calculated via the 2^−ΔCt^ method;^2^ the levels were normalized to those of the housekeeping gene *GAPDH*.^2^^,^^30^

### Elasticity analysis of the gels using both unconfined and confined compression tests

**Figure 3:**
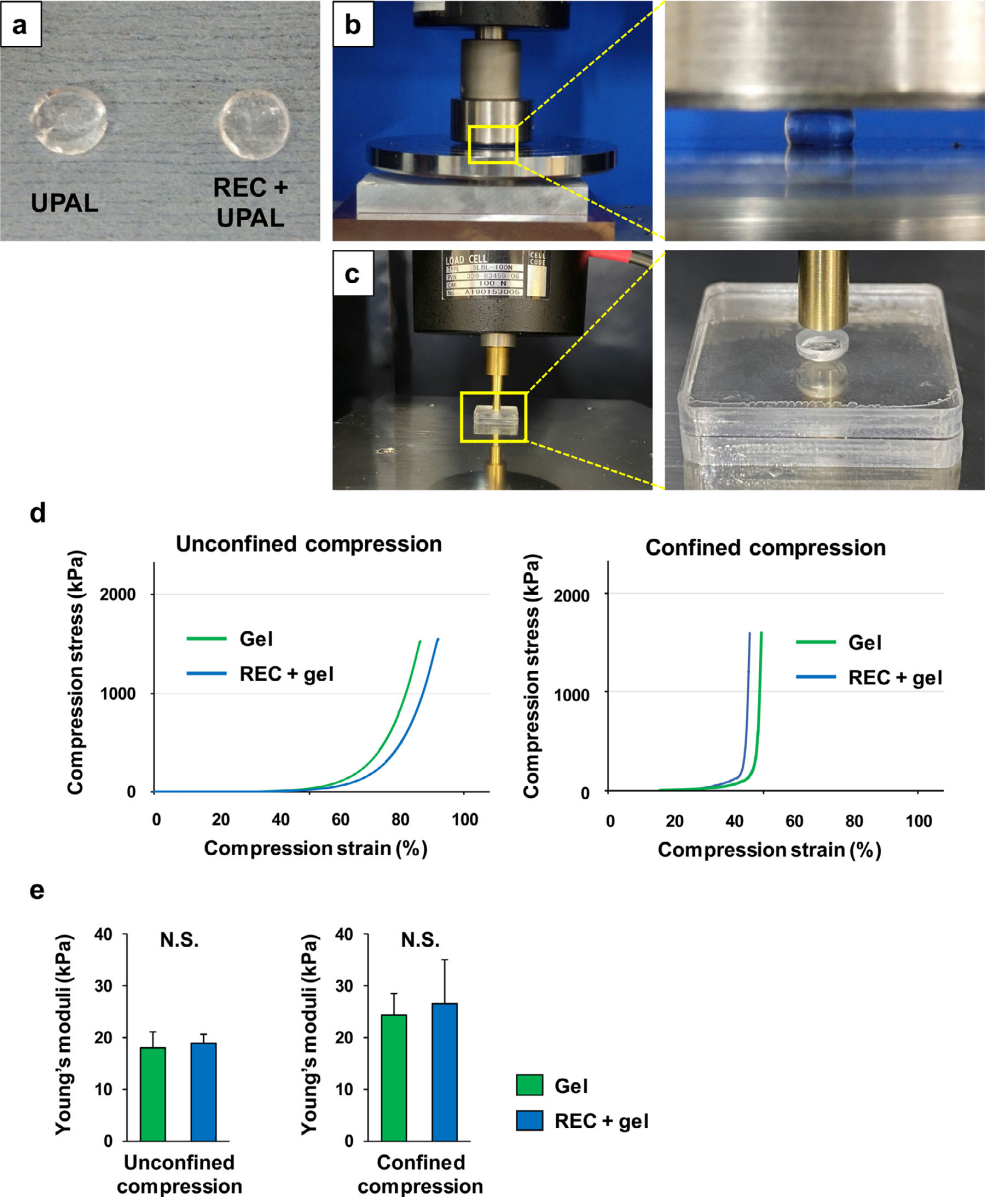
Elastic ratios of two types of gels. **(a)**: Formation of disc-shaped ultra-purified alginate (UPAL) and rapidly expanding clone (REC) + UPAL gels after CaCl_2_-induced gelation (diameter, 4·5 mm; thickness, 2 mm). **(b, c)**: Tensile-compressive mechanical testing device for unconfined compression test (**b**) and confined compression test (**c**). In the confined compression test, the disc-shaped gels were fitted within the confining chamber made of acrylic resin which had a cylindrical hole (diameter, 4·5 mm; depth, 3 mm), and pressed with a brass hammer (**c**). The sample was compressed at a constant rate of 0.5 mm/min. **(d)**: Stress-strain curve. Young's moduli were calculated according to the slope of the tangent between compression values of 10–20% compression. Four replicates were tested, and representative images are shown. **(e)**: Young's moduli of two types of gels. Data indicate mean ± SD values (n = 4). Significant differences were assessed by the Student's *t*-test, following Welch's test. N.S., not significant.

The mechanical properties of the UPAL and REC-UPAL gels were evaluated using both unconfined and confined compression tests. We prepared two types of disc-shaped gels with a diameter of 4·5 mm and thickness of 2 mm (Figure 3a). In the confined compression test, the disc-shaped gels were fitted within the confining chamber made of acrylic resin with a cylindrical hole (diameter, 4·5 mm; depth, 3 mm), and pressed with a brass hammer. The samples were placed in a tensile-compressive mechanical tester (Autograph AG-X; Shimadzu Corporation, Kyoto, Japan) and compressed at a constant rate of 0·5 mm/min using a 100 N load cell, until the gels collapsed (Figure 3b and c).^3^^,^^15^ Young's moduli were calculated based on the obtained stress-strain curve (Figure 3d) using an approximate straight line between compression values of 10–20% (n = 4 gels per group) (Figure 3e).^3^^,^^15^^,^^31^^,^^32^ These tests did not mimic the IVD; however, Young's modulus was used to assess the mechanical properties of alginate gels with and without cells.^3^^,^^15^^,^^32^ or NP of the human IVD.^31^

### *In vivo* study in a sheep model of degenerated IVD

**Figure 4:**
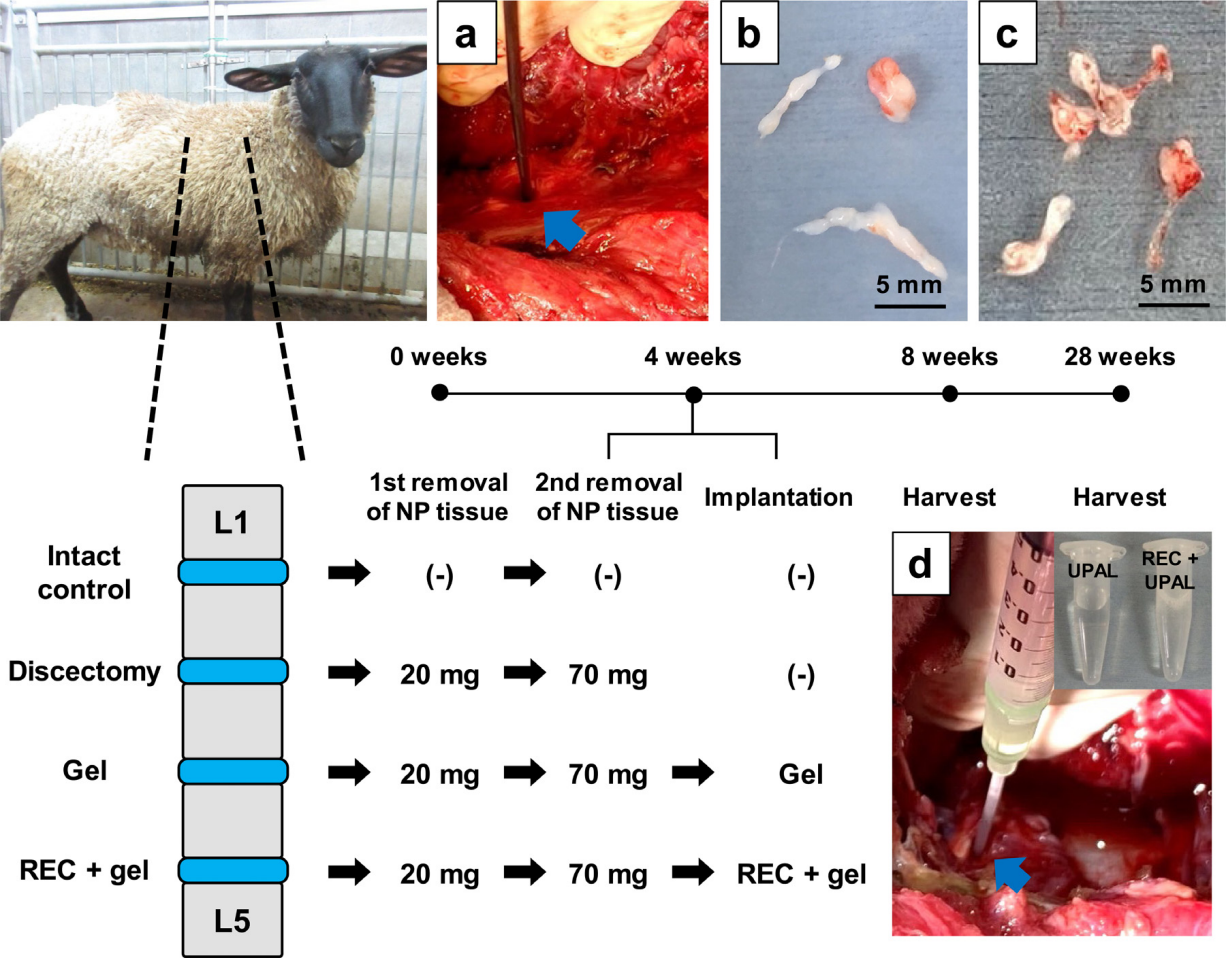
Time schedule and treatment details for each group. **(a, b)**: At the initial operation, 20 mg of fresh (nucleus pulposus) NP tissue was removed from treatment intervertebral discs (IVDs) to establish the severely degenerated IVD model. **(c)**: Four weeks after the first operation, 70 mg of degenerated NP tissue was further removed from the degenerated IVDs. **(d)**: After the removal of the degenerated NP, (ultra-purified alginate) UPAL or REC + UPAL solution was implanted into the IVD cavity.

In this study, all procedures involving the sheep model were performed in a medical product GLP-adapted laboratory (Hamri Co., Ltd.).^3^ Fourteen male, Suffolk sheep (2-year old, weighing 40–60 kg) were used for the qualitative analysis of IVD degeneration.^3^ A total of 56 IVDs were randomly allocated to the Intact control (4 weeks, n = 6; 24 weeks, n = 6), Discectomy (4 weeks, n = 6; 24 weeks, n = 6), Gel (4 weeks, n = 8; 24 weeks, n = 8), and REC + gel (4 weeks, n = 8; 24 weeks, n = 8) groups. First, the NP tissue removal surgery was performed to create a severely degenerated IVD model. Anesthesia induction was performed via the intramuscular injection of a 4:1 mixture of ketamine (0·2 mg/kg) and xylazine (20 mg/kg), at a rate of 0·5 mL/kg, and maintenance of anesthesia was achieved via inhalation anesthesia (isoflurane). The operation was performed through a right lateral retroperitoneal approach, and vertebral bodies and IVDs from L1 to L5 were exposed. A solid cancellous screw (ZIMMER BIOMET, Warsaw, IN, USA) was inserted into the L2 vertebral body as a vertebral landmark. In the Discectomy, Gel, and REC + gel groups, 20 mg of NP tissue was removed after AF incision (5 × 3 mm), and IVD degeneration was induced (Figure 4a and b).^3^^,^^8^

### Removal of degenerated NP tissue and implantation

Four weeks after the initial surgery, the vertebral bodies and IVDs were exposed using the same approach. We further extracted 70 mg of NP tissues from the degenerated IVDs obtained from the three treatment groups to create IVD cavities after an AF incision, as described above (Figure 4c). After discectomy, IVD cavities were implanted with 110–120 µL of 2% UPAL solution in the Gel groups, and 110–120 µL of a mixture of RECs and UPAL solution (final concentration, 1×10^6^ cells/mL) was implanted into the REC + gel group (Figure 4d).^2^ Immediately, 102 mM CaCl_2_ solution was injected onto the surface of the solution, and gelation was confirmed after 5 min. After 4 and 24 weeks of implantation, the sheep were euthanized using pentobarbital and their lumbar spines were removed.^3^

### MRI analysis

T2-weighted midsagittal section images were obtained using a 3·0-T MR scanner (MAGNETOM Prisma; Siemens, Munich, Germany). To evaluate the change in the signals of treated IVDs, we scored the degree of IVD degeneration using the Pfirrmann classification method,^36^ which involves five grades (1: normal to 5: highly degenerated). In addition, using Analyze 14·0 software (AnalyzeDirect, Overland Park, KS, USA), the MRI index values (the product of the mean signal intensity of the NP and the NP area) were measured to quantify the radiance of the NP tissue. We evaluated the relative MRI index, which is the ratio of the MRI index in the Intact control group, in all three treatment groups.^2^^,^^3^^,^^15^^,^^33^^,^^34^ The disc height index (DHI), which is the ratio of the disc height to the height of the adjacent vertebral body on the cranial side, was also measured.^4^^,^^38^ We determined the relative DHI, which is a percentage value of the DHI of the Intact control group (Supplementary Figure 2).

### Histological analysis

After MRI imaging, samples were fixed in 10% formaldehyde, demineralized with 10% EDTA (pH 7·5), and embedded in paraffin. Sagittal 5-micrometer-thick paraffin sections were deparaffinized with xylene for 20 min, treated with alcohol for 15 min, washed with water for 5 min, rinsed with distilled water for 30 s, and then stained with H&E and safranin-O. The degrees of IVD degeneration were scored from 0 (normal) to 36 (highly degenerative), using the modified Boos’ classification system.^3^^,^^39^^,^^40^

### IHC analysis

IHC was performed to determine the expression of Type II and I collagen in the IVDs. Sections were deparaffinized in xylene and treated with 0·1% trypsin for 30 min for antigenic activation. Then, sections were treated with 3% H_2_O_2_ in methanol for 10 min, followed by protein blocking for 30 min using the Protein Block Serum-Free solution (DAKO, Agilent, Santa Clara, CA, USA). Goat anti-Type I Collagen (1:40; Southern Biotech, Birmingham, AL, USA; Cat# 1310-01, RRID: AB_2753206) was used with anti-Type I collagen antibodies and anti-hCL (II) and purified IgGs (1:400; Kyowa Pharma Chemical Co., Ltd., Toyama, Japan; Cat # F-57) were used with anti-Type II collagen antibodies as the primary antibodies. The sections were incubated at room temperature for 1 h for the primary antibody response. After washing cells with PBS, the EnVision+ System-HRP Labeled polymer anti-mouse (DAKO; Cat # K4001) for Type II collagen and Histofine® Simple Stain Max PO (G) (Nichirei Biosciences, Tokyo, Japan; Cat # 414162F) for Type I collagen were used as the secondary antibodies. The sections were incubated at room temperature for 30 min for the secondary antibody response. Finally, sections were stained with DAB (DAKO) and hematoxylin. The number of Type II and I collagen-positive cells was determined in five randomly selected fields, and the positive cell percentages in total cells were calculated.^2^^,^^3^^,^^15^

### Tumorigenesis analysis

To evaluate the tumorigenesis of implanted and existing cells in implanted IVDs, the following aspects were evaluated: (i) invasive growth, (ii) karyomitosis, (iii) binucleate cells, and (iv) nucleoli. Using H&E-stained specimens in the Intact control and REC + gel groups during the 24-week evaluation period, the total number of positive cells was determined in 15 randomly selected visualization fields (15 visualization fields at a magnification of 400×; 26·5 visualization fields, 5 mm^2^ in total), and we calculated the numbers per square millimeter.

### Statistical analysis

Sample sizes for quantitative data were determined by power analysis, using the Tukey-Kramer test, with an α level of 0·05 and power of 0·8. Statistical analyses were performed using the JMP Pro version 14·0 software (SAS Institute, Cary, NC, USA), and values were considered significant if *p* < 0·05. All data are presented as mean ± SD values. One-way analysis of variance (ANOVA) and the Tukey-Kramer post hoc test were conducted for multigroup comparisons. Student's *t*-test or Mann-Whitney *U* test and Welch's test were used for two-group comparisons. We performed sample randomization and were blinded to the samples being tested.

### Role of the funding source

The funders had no role in study design, data collection, data analysis, data integration, or report preparation.

## Results

### Comparison of RECs and commercially available human BMSCs

Details of the results of the stability checks for RECs and commercially available human BMSCs prior to and after their incorporation into the gel are shown in Tables 3 and 4.

**Table 3:** Comparison of RECs and commercially available human BMSCs.

	RECs	BMSCs	*P*-value
Cell viability confirmation test (%)	94·7 ± 1·1	94·0 ± 1·8	0·6087
Cell size confirmation test (%)	34·9 ± 1·0	40·5 ± 3·0	0·0366
Cell doubling time (hr)	34·5 ± 5·4	51·9 ± 2·6	0·0074
Cell surface antigen confirmation test (%) CD90(upper) and CD45(lower)	99·6 ± 0·1	98·3 ± 0·2	0·0004
	0·0 ± 0·0	0·03 ± 0·02	0·0907

Data are means ± SD (n = 3). *P*-value was determined by Student's *t*-test.

**Table 4:** Comparison of RECs and commercially available BMSCs after 1 week culture.

		RECs	BMSCs	*P*-value
Cell doubling time (hr)	2D plate culture	54·2 ± 14·9	44·7 ± 1·5	0·3339
	3D culture within UPAL gel	57·9 ± 14·5	54·2 ± 5·0	0·6996
CD90 (%)	2D plate culture	99·6 ± 0·1	99·0 ± 0·1	0·0039
	3D culture within UPAL gel	92·8 ± 6·6	49·0 ± 4·6	0·0007
CD45 (%)	2D plate culture	0·0 ± 0·0	0·05 ± 0·02	0·0038
	3D culture within UPAL gel	0·07 ± 0·06	0·03 ± 0·02	0·3221

Data are means ± SD (n = 3). *P*-value was determined by Student's *t*-test.

The ratio of PI-unstained cells to the total number of cells was defined as the cell viability. There was no significant difference in the ratios between RECs and BMSCs (*p* = 0·6087, Student's *t*-test). The coefficient of variation (CV) of the forward scattered light proportional to the cell diameter was calculated to evaluate the cell size uniformity. The %CV value was significantly lower in RECs than in BMSCs (*p* = 0·0366, Student's *t*-test). Cell proliferative capacity was evaluated by calculating the Td. The Td were significantly shorter in RECs than in BMSCs (*p* = 0·0074, Student's *t*-test). Cell surface antigen confirmation test showed that the percentage of CD90-positive cells was significantly higher in RECs than in BMSCs (*p* = 0·0004, Student's *t*-test). There was no significant difference in CD45-positive cells between the groups (*p* = 0·0907, Student's *t*-test) (Table 3).

We further compared RECs and commercially available BMSCs after 1 week of culture. There was no significant difference between RECs and BMSCs in terms of the Td after both 2D and 3D cultures (*p* = 0·3339, *p* = 0·6996, Student's *t*-test). However, the percentages of CD90-positive cells were significantly higher among RECs than among BMSCs after both 2D and 3D cultures (*p* = 0·0039, *p* = 0·0007, Student's *t*-test). Regarding the CD45-positive cells, the percentage was significantly lower in RECs than in BMSCs after 2D culture (*p* = 0·0038, Student's *t*-test); however, there was no significant difference between RECs and BMSCs after 3D culture (*p* = 0·3221, Student's *t*-test) (Table 4).

### Co-culture of RECs and human NPCs

We investigated the effects of co-culture of RECs with human NPCs and compared them with co-culture of commercially available human BMSCs and human NPCs of different cell types. We measured the expression of NPC markers, including HIF-1α, GLUT-1, and brachyury; growth factors, including CDMP-1, TGF-β, and IGF-1; and ECM components, including Type II collagen and aggrecan, in these cells via real-time qRT-PCR.^2^^,^^9^^,^^29^^,^^30^

The expression levels of three NPC markers, HIF-1α, GLUT-1, and brachyury, were significantly higher in NPCs co-cultured with RECs than those in control NPCs, mono-cultured NPCs, and NPCs co-cultured with commercial BMSCs. GLUT-1 expression levels were significantly higher in NPCs co-cultured with commercial BMSCs than those in control NPCs (Figure 2c–e). The expression levels of CDMP-1 and IGF-1 were significantly increased in NPCs co-cultured with RECs compared with those in control NPCs, mono-cultured NPCs, and NPCs co-cultured with commercial BMSCs. The TGF-β expression levels were significantly increased in NPCs co-cultured with RECs compared with those in control and mono-cultured NPCs. NPCs co-cultured with commercial BMSCs showed a significant increase in CDMP-1 and IGF-1 expression compared with control NPCs and mono-cultured NPCs (Figure 2f–h). The expression levels of Type II collagen and aggrecan were significantly higher in NPCs co-cultured with RECs than those in control and mono-cultured NPCs; their levels in NPCs co-cultured with commercial BMSCs were also significantly higher than those in control and mono-cultured NPCs (Figure 2i and j).

The expression levels of all three NPC markers in co-cultured RECs were significantly higher than those in control and mono-cultured RECs (Figure 2c–e). The expression levels of CDMP-1, TGF-β, and IGF-1 were also significantly increased in co-cultured RECs compared with those in control and mono-cultured RECs (Figure 2f–h). Similarly, the expression levels of Type II collagen and aggrecan were significantly higher in co-cultured RECs than those in control and mono-cultured RECs (Figure 2i and j).

### Effects of RECs embedded in the UPAL gel

Both unconfined and confined compression tests were performed to evaluate and compare the mechanical properties of the REC-UPAL and UPAL gels, as described previously.^3^^,^^15^^,^^31^^,^^32^ We prepared two types of cylindrical gel samples (diameter: 4·5 mm, thickness: 2 mm) and calculated the ratio of longitudinal compressive stress to strain (Figure 3a–c). At compression levels of 10–20%, the Young's moduli were 18·0 ± 3·5 kPa and 18·8 ± 2·1 kPa for the UPAL and REC-UPAL gels in the unconfined test, respectively, and 24·3 ± 4·2 kPa and 26·5 ± 8·5 kPa for the UPAL and REC-UPAL gels in the confined test, respectively; no significant differences in mechanical properties were observed between the two groups (*p* = 0·6942 in unconfined test and *p* = 0·6623 in the confined test; *p* values measured with Student's *t*-test) (Figure 3d and e). Additionally, these two values were comparable to those of healthy human NP tissues.^3^^,^^15^^,^^31^

### Combination of RECs and UPAL gel facilitates IVD regeneration

**Figure 5:**
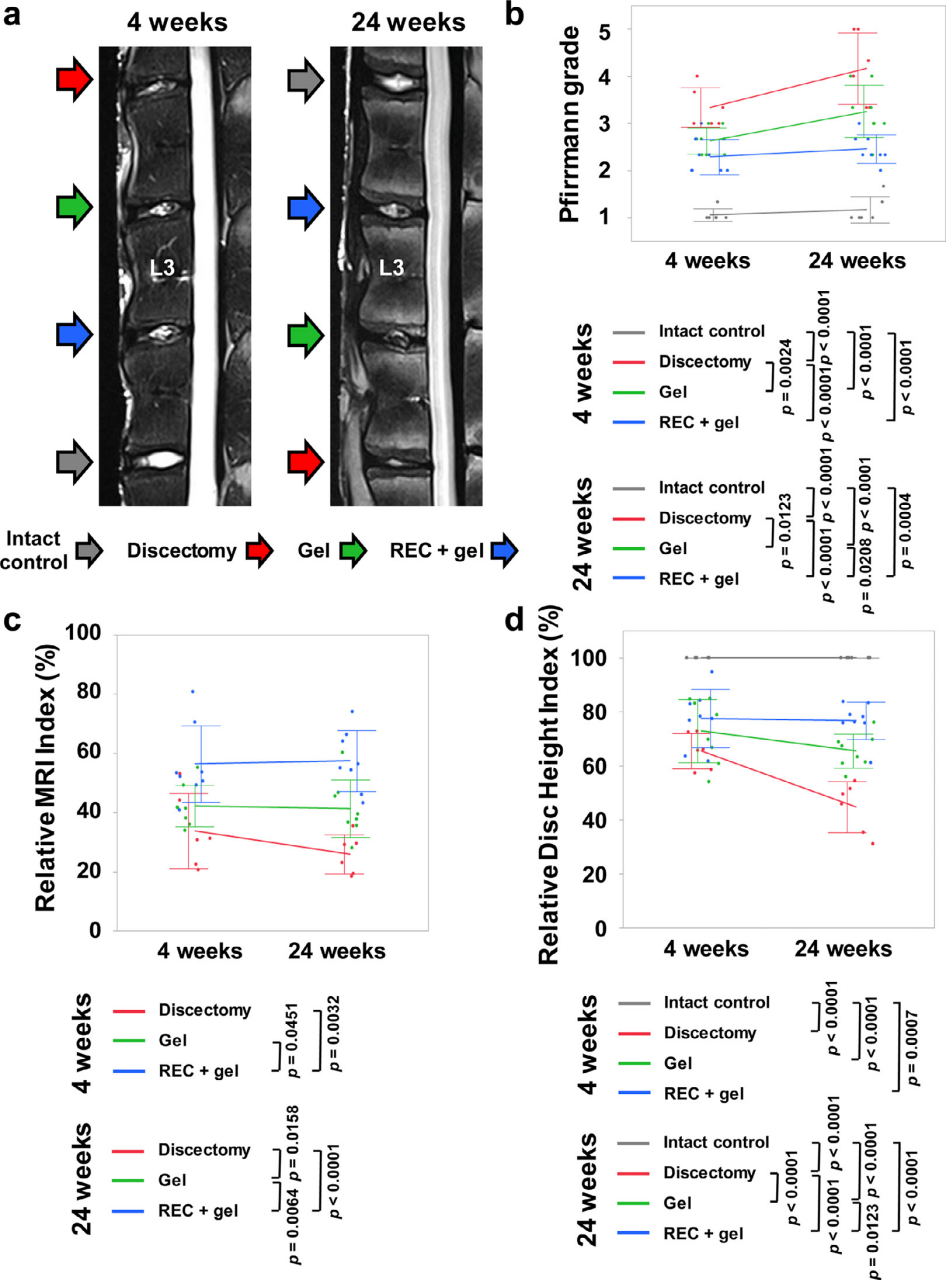
Magnetic resonance imaging (MRI) assessment of the treated intervertebral discs (IVDs) at 4 and 24 weeks post-implantation. **(a)**: T2-weighted, midsagittal images of IVDs in sheep, at 4 and 24 weeks after surgery. Images are representative of 6 or 8 replicates. **(b)**: Pfirrmann grade of IVD degeneration. **(c)**: MRI index (Nucleus pulposus (NP) area × average signal intensity) values for degenerative alterations in the NP. Numerical values are expressed as percentages relative to the values for intact control IVDs. **(d)**: Disc height index for IVD treatment. Numerical numbers are shown as percentages relative to those of intact control IVDs. Data represent mean ± SD values (Intact control, n = 6; Discectomy, n = 6; Gel, n = 8; REC + gel, n =8). Significant differences were assessed by one-way ANOVA with post hoc analysis using the Tukey-Kramer test.

The degenerative changes in treated IVDs were evaluated using T2-weighted midsagittal section images obtained via MRI (Figure 5a). Based on the Pfirrmann scores, the grades of the Gel group were significantly lower than those of the Discectomy group at 24 weeks (*p* = 0·0123, Tukey–Kramer test), and the grades of the REC + gel group were significantly lower than those of the Discectomy and Gel groups at both 4 and 24 weeks (4 weeks; *p* < 0·0001 (vs Discectomy), 24 weeks; *p* < 0·0001, *p* = 0·0208, Tukey–Kramer test) (Figure 5b).

The MRI index was significantly higher in the REC + gel group than in the Discectomy and Gel groups at 4 weeks (*p* = 0·0032, *p* = 0·0032, Tukey–Kramer test). At 24 weeks, the MRI index of the Gel group was significantly higher than that of the Discectomy group (*p* = 0·0158, Tukey–Kramer test) and that of the REC + gel group was significantly higher than that of the Discectomy and Gel groups (*p* < 0·0001, *p* = 0·0064, Tukey–Kramer test) (Figure 5c).

Additionally, the disc heights of treated IVDs were measured using MRI images. The DHI values of the three treated groups were significantly lower than those of the Intact control group at both 4 and 24 weeks. Although there was no significant difference among the three groups 4 weeks after implantation, the DHI of the Gel group was significantly higher than that of the Discectomy group (*p* < 0·0001, Tukey–Kramer test) and that of the REC + gel group was significantly higher than that of the Gel and Discectomy groups 24 weeks after implantation (*p* = 0·0123, *p* < 0·0001, Tukey–Kramer test) (Figure 5d).

**Figure 6:**
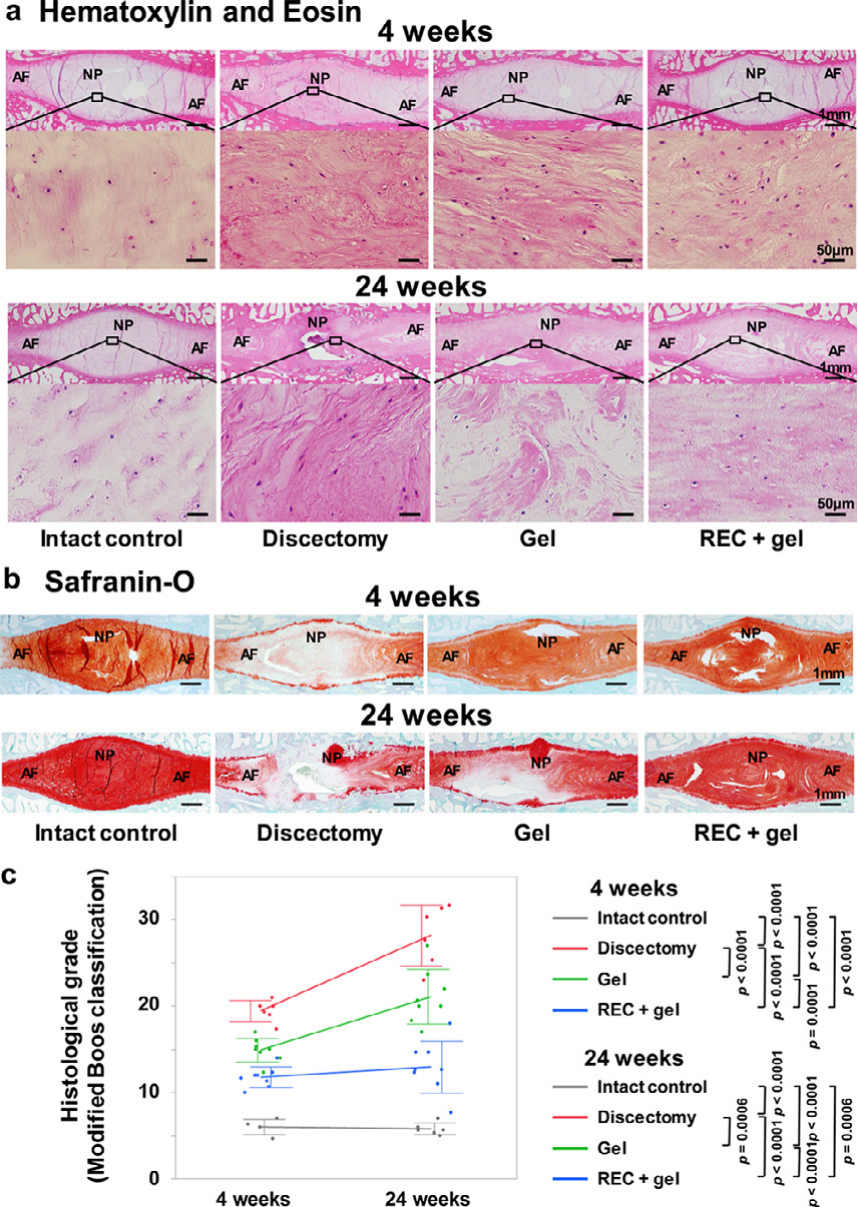
Histological evaluation of the treated intervertebral discs (IVDs) 4 and 24 weeks after implantation. **(a, b)**: Representative midsagittal sections of treated IVDs stained via hematoxylin & eosin (H&E) or safranin-O staining (Intact control, n = 6; Discectomy, n = 6; Gel, n = 8; REC + gel, n = 8). Scale bar = **(a)**: 50 μm (second and fourth sections from the top) or 1 mm (first and third sections from the top), **(b)**: 1 mm. **(c)**: Histological grades determined via modified Boos’ classification. Data represent mean ± SD values. Significant differences were assessed by one-way ANOVA with the post hoc Tukey-Kramer test. AF, annulus fibrosus; NP, nucleus pulposus.

Histological analysis was performed using H&E and safranin-O staining. At both 4 and 24 weeks in the Intact control group, the IVDs exhibited a spindle shape, no fibrotic changes in the NP tissue, concentric AF, and uniform staining with safranin-O. Although IVD tissue structures were preserved effectively in the REC + gel group, the tissues were mildly crushed and exhibited many fibrotic changes; a partial loss of safranin-O staining was observed in the Gel group. Considerable amount of scar tissue, several fibrotic changes, tissue loss, and endplate collapse and destruction were observed in the Discectomy group (Figure 6a and b). Histological grades determined based on the modified Boos’ classification^3^^,^^39^^,^^40^ in the Gel group were significantly lower than those in the Discectomy group at both 4 and 24 weeks (*p* < 0·0001, *p* = 0·0006, Tukey–Kramer test) and were significantly lower in the REC + gel group than those in the Discectomy and Gel groups at both 4 and 24 weeks (4 weeks; *p* < 0·0001, *p* = 0·0001, 24 weeks; *p* < 0·0001, *p* < 0·0001, Tukey–Kramer test) (Figure 6c).

**Figure 7:**
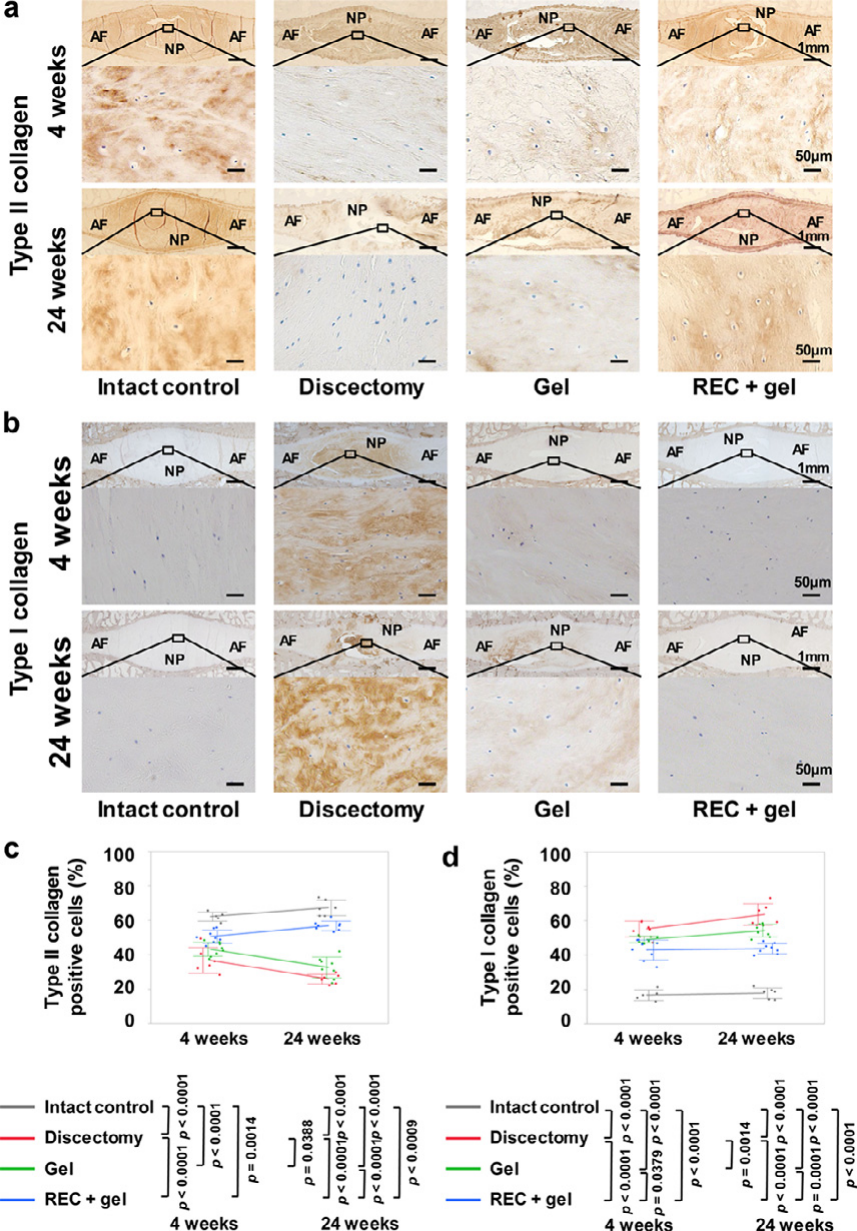
Type II or I collagen-positive cells in the treated intervertebral discs (IVDs) 4 and 24 weeks after implantation. **(a, b)**: Representative midsagittal sections of treated IVDs stained for Type II collagen or Type I collagen (Intact control, n = 6; Discectomy, n = 6; Gel, n = 8; REC + gel, n = 8). Scale bar = 1 mm (first and third lines) or 50 μm (second and fourth lines). **(c, d)**: Percentages of Type II or I collagen positive cells to total cells in treated IVDs. Data represent mean ± SD values. Significant differences were assessed by one-way ANOVA with the post hoc Tukey-Kramer test. AF, annulus fibrosus; NP, nucleus pulposus.

The expression levels of Type II and I collagen in treated IVDs were assessed by IHC (Figure 7a and b). The percentages of Type II and I collagen-positive cells—in the total number of cells—in five randomly selected visualization fields were determined in NP tissues.^2^^,^^3^^,^^15^ Type II collagen is an essential ECM component in NP tissues and is replaced by Type I collagen with the progression of degeneration. Analysis of Type II collagen showed that NP tissues were uniformly stained in the Intact control group, but a slight decrease in intensity was observed in the REC + gel groups. Scattered non-stained areas were observed in the Gel groups and broadly non-stained areas were observed in the Discectomy group. The percentage of Type II collagen-positive cells was significantly higher in the REC + gel group than that in the Discectomy group at the 4-week evaluation period (*p* < 0·0001, Tukey–Kramer test) and was significantly higher than that in the Discectomy and Gel groups at the 24-week evaluation period (*p* < 0·0001, *p* < 0·0001, Tukey–Kramer test). The percentage of these cells were significantly higher in the Gel group than those in the Discectomy group at 24 weeks (*p* = 0·0388, Tukey–Kramer test) (Figure 7c). However, the percentages of Type I-collagen-positive cells were significantly lower in the REC + gel group than those in the Discectomy and Gel groups at both 4 and 24 weeks (4 weeks; *p* < 0·0001, *p* = 0·0379, 24 weeks; *p* < 0·0001, *p* = 0·0001, Tukey–Kramer test). In addition, the percentage of these cells was significantly lower in the Gel group than in the Discectomy group at 24 weeks (*p* = 0·0014, Tukey–Kramer test) (Figure 7d).

### Effects of REC + gel implantation on IVDs

Tumorigenesis was analyzed in histological specimens in the Intact control and REC+ gel groups during the 24-week evaluation period. Invasive growth, along with karyomitosis, was assessed in all specimens of both groups, and the number of binucleate cells and nucleoli per square millimeter was determined. Invasive growth, karyomitosis, and nucleoli were not observed in both groups. The number of binucleate cells in the Intact control and REC + gel groups was 0·03 ± 0·07 and 0·23 ± 0·25, respectively, i.e., the values were < 1 /mm^2^ and no significant differences were observed between the groups (*p* = 0·3311, Mann-Whitney *U* test) (Supplementary Table 2).

## Discussion

Although several studies have reported the regenerative ability of various BMSCs in IVDs and the effect of BMSCs incorporated in various gel-types and model systems has been shown earlier,^2^^,^^15^^,^^43^, ^44^, ^45^ further biomedical advancement is needed to achieve better clinical safety and efficacy in applied regenerative medicine. BMSCs with consistently high cell quality are required for clinical applications. The use of RECs, characterized according to the International Society for Cellular Therapy (ISCT) criteria for human BMSCs,^46^^,^^47^ represents a notable advantage of this study, as the cell source yields high-quality BMSCs with minimal batch-to-batch variance.^18^ In this study, excellent characteristics of RECs were shown in terms of cell proliferative capacity, cell size uniformity, and the expression of cell surface antigens compared to those of commercially available human BMSCs. In addition, the present study demonstrates that the expression levels of NPC markers, growth factors, and ECM components were significantly increased in the 3D co-culture of human NPCs and RECs compared with those observed in the 3D co-culture of NPCs with commercially available BMSCs. The efficacy of the combination of RECs and UPAL gel was observed at the site of IVD degeneration in a sheep lumbar spine model. Though the UPAL gel alone delayed IVD degeneration (compared to that following discectomy) the combination of RECs and gel enhanced IVD regeneration more effectively.

In this study, the expression levels of all three NPC markers in co-cultured RECs were significantly higher than those in the control and mono-cultured RECs, indicating that the co-culture of RECs with NPCs results in the differentiation of RECs into NPCs, thereby improving ECM component production in both cell types. This is consistent with the findings of our previous—and other—*in vitro* studies, which revealed the crosstalk between NPCs and BMSCs through growth factor production^2^^,^^30^^,^^48^^,^^49^ and enhanced ECM synthesis.^2^^,^^9^^,^^30^^,^^35^^,^^50^ Similar results were observed in our previous study in rabbits, which revealed increased expression of NPC markers after the implantation of BMSCs encapsulated in the UPAL gel and increased production of ECM compared with that observed following discectomy without the implantation of BMSCs encapsulated in the UPAL gel.^2^ Based on the results of present and previous studies,^2^^,^^30^^,^^48^^,^^49^^,^^50^, ^51^, ^52^ the following are the possible mechanisms underlying IVD regeneration in response to the implantation of RECs encapsulated in the UPAL gel: (1) RECs can be implanted in IVD defects through gel encapsulation (gel encapsulation is not a mechanism of IVD regeneration and just the technique used in the study); (2) RECs produce growth factors and ECM components, thereby activating the existing NPCs (paracrine mechanism); (3) NPCs produce more ECM and growth factors; (4) consequently, RECs directly differentiate into NPCs; (5) finally, RECs and NPCs have positive intercellular feedback loop resulting in IVD regeneration.^2^

The biomaterials/hydrogels used for IVD restoration should be biologically and mechanically suitable. Hence, comprehensive biomechanical investigations need to focus on the clinically relevant loads in the context of daily human activity, which are thought to be essential for the regulation of IVD repair.^3^ Presently, there are no commercially available soft biomaterials used in routine clinical practice to fix discectomy-associated defects, likely because of the risk that high intradiscal pressures might result in their ejection.^3^ Food and Drug Administration (FDA) guidelines or American Society for Testing and Materials (ASTM) International standards are not currently available for evaluating the adhesion of hydrogel to IVD tissues.^53^^,^^54^ However, an *ex vivo* experiment could be used to investigate the mechanical properties of a hydrogel. Here, we selected a sheep lumbar spine model for implanting the candidate hydrogel into a preclinical animal model, because the biomechanics and geometry of lumbar IVDs are comparable to those of humans.^3^^,^^4^^,^^41^ We had previously shown that the UPAL gel implanted into cadaveric sheep lumbar IVDs after discectomy exhibited appropriate biomechanical characteristics, and did not result in material protrusion and require suturing of the AF after discectomy in sheep spines.^3^ In addition to six-axis static loading tests, sinusoidal dynamic axial loading (± 300 N) tests were applied for 1000 cycles, in the context of daily human activity, mimicking how an individual stoops down 10 times per day, and the approximate period after which the gel disappears (100 days) was determined.^3^ Although there have been some studies on hydrogels, wherein dynamic mechanical evaluations were performed immediately after the injection of the gel into the IVD using a human or large animal cadaveric model,^55^^,^^56^ to our knowledge, none of the previous studies investigating GMP-compliant gels have addressed this issue.^3^ In this study, the results of both the unconfined and confined compression tests revealed that there were no significant differences in the Young's moduli between the UPAL and REC-UPAL groups, indicating that the RECs embedded in the UPAL gel did not alter the mechanical characteristics of the gel.^15^ This could be expected given the low concentration of BMSCs, which essentially are non-adherent and therefore non-contracting. Also, cells generally do not adhere to alginate. It should, however, be considered that the REC concentration utilised in this study (1 × 10^6^ cells/ml) was based on the results of a previous study that used a canine IVD degeneration model to determine the optimal BMSC number for transplantation; ^57^ the results showed that both the structural microenvironment and ECM were maintained following the transplantation of 1 × 10^6^ BMSCs to the canine IVDs compared to that of 1 × 10^7^ BMSCs. Accordingly, we did not attempt to apply higher concentrations of RECs to IVDs, greatly beyond the current concentration, and did not test the effect of the cells on the mechanical properties of the gel at a higher concentration. Thus, owing to its crucial ability to bring about rapid healing, the combination of the UPAL gel with RECs at the current concentration offers clinical advantages in terms of preventing cell leakage without suturing the AF.^15^

Many studies have reported on the discectomy of non-degenerated IVDs in a large animal model.^3^^,^^4^^,^^8^^,^^12^, ^13^, ^14^^,^^42^^,^^45^ However, to the best of our knowledge, this is the first study to report on a discectomy for degenerated IVDs in a large animal model. MRI and histological evaluation in animals implanted (and not implanted) with UPAL gel indicates that IVD degeneration was delayed following the implantation of UPAL gel alone. Moreover, the combination of RECs and UPAL gel prevented degeneration even more effectively. There was no significant difference in the number of binucleate cells between the Intact and REC + gel groups, suggesting that the combination of RECs with the gel did not induce tumorigenesis. In addition, IHC suggests that the combined use of RECs and gel enhances ECM synthesis in the degenerated NP tissue, a phenomenon that is essential for the functioning of native IVD, and prevents the progression of IVD degeneration by downregulating the production of Type I collagen. These results are consistent with our previous results—obtained using a rabbit model of IVD degeneration—and demonstrate that BMSCs encapsulated in the UPAL gel prevented degeneration more effectively.^2^^,^^15^ The previous results also showed that the histological grading score for degeneration was lower after BMSC + UPAL gel implantation, compared to that in animals not subjected to discectomy, in which degenerated IVDs were created via AF needle puncture. This suggests that BMSC implantation resulted in IVD regeneration.^2^^,^^15^ Taken together, our present and previous results indicate that the implantation of RECs encapsulated in the UPAL gel enhances IVD regeneration *in vivo*.^2^^,^^15^

The present study provided valuable insights into the translational approach of combining GMP-compliant BMSCs with soft biomaterials to enhance IVD regeneration post-discectomy; however, a few limitations need to be addressed. First, since unconfined compression test is the representative mechanical testing method for measuring the mechanical properties of cylindrical hydrogel samples^3^^,^^15^^,^^32^ and as we previously reported the mechanical properties of a UPAL gel with and without cells using this test,^3^^,^^15^ in the present study, we conducted the same test. However, the characterization of the hydrogel with Young's modulus based on results of the simple compression test is insufficient because hydrogels and soft tissues are poro-visco-elastic tissues with evident time-dependent properties. To investigate the complexity of the material, aggregate modulus and permeability based on confined compression tests should be further assessed using human NP.^58^ Second, the animal models of IVD degeneration used in the present study were generated using sheep IVDs; therefore, the surgical procedure of discectomy was not strictly replicated in clinical settings. Although discectomy was performed in the sheep model through the antero-lateral approach, clinical discectomy is typically performed through the posterior approach.^2^, ^3^, ^4^ However, sheep models subjected to discectomy performed through the antero-lateral approach with subsequent UPAL gel implantation exhibited IVD defect repair. These results were similar to those obtained in the present model.^3^ Additionally, discectomy of cadaveric sheep lumbar IVDs through the posterior approach demonstrated sufficient biomechanical advantages, and did not result in material protrusion^3^. No material protrusion was observed in a clinical trial, in which the gel was implanted into the defect after discectomy in 40 young patients (data not shown).^28^ Therefore, the current results might be applicable to humans. Third, although the AF shows a material with a highly complex structure and has a pivotal role in the containment of the NP and/or its substitutes, a detailed evaluation of AF regeneration was not performed. It appears that the UPAL gel is capable of containing the alginate within, also under *in vivo* conditions, however, this requires an attachment of the gel to the adjacent vertebrae and the remainder of the AF. Because IVD degeneration is characterized by the degradation of the NP ECM and our previous study showed no UPAL gel protrusion from the cadaveric sheep IVDs,^3^ the current study concentrated principally on NP preservation/regeneration.^2^^,^^3^^,^^15^ However, the present study evaluated the morphological changes in the AF structure based on a histological scale.^2^^,^^3^^,^^39^ Last, the intervention has been carried out in sheep in acute and sub-acute phases of IVD disruption, and therefore the efficacies shown here may be different from those in chronic cases, which were not the subject of this model. However, as most patients will present with chronic stages of IVD, the efficacy of the implants in such cases will require further evaluation. In addition, though the pre-degeneration of the IVD carried out in this study could be considered an improvement over the investigation of non-degenerated IVDs undertaken in previous studies, this improvement of the model was not quantified. Furthermore, it is not completely clear whether a four-week degeneration period is sufficient for obtaining a representative model of the human IVD after herniation. It is known that the AF of the human IVD is damaged over the entire posterior part, while there is only a protrusion on one posterior-lateral part. The posterior-contralateral region of the AF in human IVD is also damaged, unlike the AF in the degenerated sheep IVD.

## Conclusions

We demonstrate that the combination of GMP-compliant, ultra-purified, BMSCs in the form of RECs and *in situ*-forming gel enhances IVD regeneration after discectomy in a sheep model exhibiting severe IVD degeneration, without resulting in neoplastic changes, and enable the maintenance of mechanical functions after implantation. Our findings and strategy need to be validated in clinical settings in future studies, especially in an elderly cohort such as individuals with combined lumbar canal stenosis.

H.S. designed the study. D.U., K.Y., T.S., T.T. and H.S. performed experiments. D.U., K.Y., T.S., T.T., T.N., M.W. and H.S. performed data acquisition, and D.U., K.Y., T.S., T.N., M.W. and H.S. analyzed the data. D.U., D.R.L., H.S., N.I., Y.M. and H.S. interpreted the results. D.U. and H.S. wrote the manuscript. H.S. revised the manuscript. All authors had full access to all data of this study and had final responsibility for the decision to submit for publication.

## Declaration of interests

T. S. and Y. M. are employees of PuREC Co., Ltd. M. W. is an employee of Japan Tissue Engineering Co., Ltd. They do not hold any other financial interests in these companies. These companies had no role in the study design, data collection, analysis, writing of the manuscript, the decision to submit the manuscript for publication or any aspect pertinent to the study. D. U., T. S., Y. M., and H. S. are listed as inventors on a patent application related to this work. The other authors declare that they have no competing interests.

## Appendix Supplementary materials

Image, application 1

Image, application 2

## References

[1] Katz J.N. (2006). Lumbar disc disorders and low-back pain: socioeconomic factors and consequences. J Bone Joint Surg Am.

[2] Ukeba D., Sudo H., Tsujimoto T., Ura K., Yamada K., Iwasaki N. (2020). Bone marrow mesenchymal stem cells combined with ultra-purified alginate gel as a regenerative therapeutic strategy after discectomy for degenerated intervertebral discs. EBioMedicine.

[3] Tsujimoto T., Sudo H., Todoh M. (2018). An acellular bioresorbable ultra-purified alginate gel promotes intervertebral disc repair: a preclinical proof-of-concept study. EBioMedicine.

[4] Sloan S.R., Wipplinger C., Kirnaz S. (2020). Combined nucleus pulposus augmentation and annulus fibrosus repair prevents acute intervertebral disc degeneration after discectomy. Sci Transl Med.

[5] Heindel P., Tuchman A., Hsieh P.C. (2017). Reoperation rates after single-level lumbar discectomy. Spine.

[6] Sherman J., Cauthen J., Schoenberg D., Burns M., Reaven N.L., Griffith SL. (2010). Economic impact of improving outcomes of lumbar discectomy. Spine J.

[7] Kambin P., Cohen L.F., Brooks M., Schaffer J.L. (1995). Development of degenerative spondylosis of the lumbar spine after partial discectomy: comparison of laminotomy, discectomy, and posterolateral discectomy. Spine.

[8] Oehme D., Ghosh P., Shimmon S. (2014). Mesenchymal progenitor cells combined with pentosan polysulfate mediating disc regeneration at the time of microdiscectomy: a preliminary study in an ovine model. J Neurosurg Spine.

[9] Naqvi S.M., Buckley C.T. (2015). Differential response of encapsulated nucleus pulposus and bone marrow stem cells in isolation and coculture in alginate and chitosan hydrogels. Tissue Eng Part A.

[10] Noriega D.C., Ardura F., Hernández-Ramajo R. (2017). Intervertebral disc repair by allogeneic mesenchymal bone marrow cells: a randomized controlled trial. Transplantation.

[11] Bowles R.D., Gebhard H.H., Härtl R., Bonassar L.J. (2011). Tissue-engineered intervertebral discs produce new matrix, maintain disc height, and restore biomechanical function to the rodent spine. Proc Natl Acad Sci USA.

[12] Revell P.A., Damien E., Di Silvio L., Gurav N., Longinotti C., Ambrosio L. (2007). Tissue engineered intervertebral disc repair in the pig using injectable polymers. J Mater Sci Mater Med.

[13] Hussain I., Sloan S.R., Wipplinger C. (2018). Mesenchymal stem cell-seeded high-density collagen gel for annular repair: 6-week results from *in vivo* sheep models. Neurosurgery.

[14] Pennicooke B., Hussain I., Berlin C. (2018). Annulus fibrosus repair using high-density collagen gel: an *in vivo* ovine model. Spine.

[15] Ukeba D., Yamada K., Tsujimoto T. (2021). Bone marrow aspirate concentrate combined with *in situ* forming bioresorbable gel enhances intervertebral disc regeneration in rabbits. J Bone Joint Surg Am.

[16] Ura K., Yamada K., Tsujimoto T., Ukeba D., Iwasaki N., Sudo H. (2021). Ultra-purified alginate gel implantation decreases inflammatory cytokine levels, prevents intervertebral disc degeneration, and reduces acute pain after discectomy. Sci Rep.

[17] Friedenstein A.J., Deriglasova U.F., Kulagina N.N. (1974). Precursors for fibroblasts in different populations of hematopoietic cells as detected by the *in vitro* colony assay method. Exp Hematol.

[18] Mabuchi Y., Morikawa S., Harada S. (2013). LNGFR(+)THY-1(+)VCAM-1(hi+) cells reveal functionally distinct subpopulations in mesenchymal stem cells. Stem Cell Rep.

[19] Pittenger M.F., Mackay A.M., Beck S.C. (1999). Multilineage potential of adult human mesenchymal stem cells. Science.

[20] Kim J., Kang J.W., Park J.H. (2009). Biological characterization of long-term cultured human mesenchymal stem cells. Arch Pharm Res.

[21] Rombouts W.J., Ploemacher R.E. (2003). Primary murine MSC show highly efficient homing to the bone marrow but lose homing ability following culture. Leukemia.

[22] Brown C., McKee C., Bakshi S. (2019). Mesenchymal stem cells: Cell therapy and regeneration potential. J Tissue Eng Regen Med.

[23] Squillaro T., Peluso G., Galderisi U. (2016). Clinical trials with mesenchymal stem cells: an update. Cell Transplant.

[24] Morikawa S., Mabuchi Y., Kubota Y. (2009). Prospective identification, isolation, and systemic transplantation of multipotent mesenchymal stem cells in murine bone marrow. J Exp Med.

[25] Houlihan D.D., Mabuchi Y., Morikawa S. (2012). Isolation of mouse mesenchymal stem cells on the basis of expression of Sca-1 and PDGFR-α. Nat Protoc.

[26] Mabuchi Y., Matsuzaki Y. (2016). Prospective isolation of resident adult human mesenchymal stem cell population from multiple organs. Int. J. Hematol..

[27] Harada S., Mabuchi Y., Kohyama J. (2021). FZD5 regulates cellular senescence in human mesenchymal stem/stromal cells. Stem Cells.

[28] Yamada K., Kenichiro M., Ito Y.M. (2021). Exploratory clinical trial on the safety and capability of dMD-001 in lumbar disc herniation: study protocol for a first-in-human pilot study. Contemp Clin Trials Commun.

[29] Risbud M.V., Schoepflin Z.R., Mwale F. (2015). Defining the phenotype of young healthy nucleus pulposus cells: recommendations of the Spine Research Interest Group at the 2014 annual ORS meeting. J Orthop Res.

[30] Strassburg S., Richardson S.M., Freemont A.J., Hoyland JA. (2010). Co-culture induces mesenchymal stem cell differentiation and modulation of the degenerate human nucleus pulposus cell phenotype. Regen Med.

[31] Iatridis J.C., Weidenbaum M., Setton L.A., Mow VC. (1996). Is the nucleus pulposus a solid or a fluid? Mechanical behaviors of the nucleus pulposus of the human intervertebral disc. Spine.

[32] Zeng Y., Chen C., Liu W. (2015). Injectable microcryogels reinforced alginate encapsulation of mesenchymal stromal cells for leak-proof delivery and alleviation of canine disc degeneration. Biomaterials.

[33] Sudo H., Minami A. (2011). Caspase 3 as a therapeutic target for regulation of intervertebral disc degeneration in rabbits. Arthritis Rheum.

[34] Ura K., Sudo H., Iwasaki K., Tsujimoto T., Ukeba D., Iwasaki N. (2019). Effects of intradiscal injection of local anesthetics on intervertebral disc degeneration in rabbit degenerated intervertebral disc. J Orthop Res.

[35] Ouyang A., Cerchiari A.E., Tang X. (2017). Effects of cell type and configuration on anabolic and catabolic activity in 3D co-culture of mesenchymal stem cells and nucleus pulposus cells. J Orthop Res.

[36] Pfirrmann C.W., Metzdorf A., Zanetti M., Hodler J., Boos N. (2001). Magnetic resonance classification of lumbar intervertebral disc degeneration. Spine.

[37] Sato M., Uchida K., Nakajima H. (2012). Direct transplantation of mesenchymal stem cells into the knee joints of Hartley strain guinea pigs with spontaneous osteoarthritis. Arthritis Res Ther.

[38] Lü D.S., Shono Y., Oda I., Abumi K., Kaneda K. (1997). Effects of chondroitinase ABC and chymopapain on spinal motion segment biomechanics: an *in vivo* biomechanical, radiologic, and histologic canine study. Spine.

[39] Boos N., Weissbach S., Rohrbach H., Weiler C., Spratt K.F., Nerlich AG. (2002). Classification of age-related changes in lumbar intervertebral discs: 2002 Volvo Award in basic science. Spine.

[40] Reitmaier S., Kreja L., Gruchenberg K. (2014). *In vivo* biofunctional evaluation of hydrogels for disc regeneration. Eur Spine J.

[41] Reitmaier S., Schmidt H., Ihler R. (2013). Preliminary investigations on intradiscal pressures during daily activities: an *in vivo* study using the merino sheep. PLoS One.

[42] Hegewald A.A., Medved F., Feng D. (2015). Enhancing tissue repair in annulus fibrosus defects of the intervertebral disc: analysis of a bio-integrative annulus implant in an *in-vivo* ovine model. J Tissue Eng Regen Med.

[43] Wang H., Zhou Y., Huang B. (2014). Utilization of stem cells in alginate for nucleus pulposus tissue engineering. Tissue Eng Part A.

[44] Wang F., Nan L.P., Zhou S.F. (2019). Injectable hydrogel combined with nucleus pulposus-derived mesenchymal stem cells for the treatment of degenerative intervertebral disc in rats. Stem Cells Int.

[45] Omlor G.W., Lorenz S., Nerlich A.G., Guehring T., Richter W. (2018). Disc cell therapy with bone-marrow-derived autologous mesenchymal stromal cells in a large porcine disc degeneration model. Eur Spine J.

[46] Dominici M., Le Blanc K., Mueller I. (2006). Minimal criteria for defining multipotent mesenchymal stromal cells: the international society for cellular therapy position statement. Cytotherapy.

[47] Raynaud C.M., Maleki M., Lis R. (2012). Comprehensive characterization of mesenchymal stem cells from human placenta and fetal membrane and their response to osteoactivin stimulation. Stem Cells Int.

[48] Yamamoto Y., Mochida J., Sakai D. (2004). Upregulation of the viability of nucleus pulposus cells by bone marrow-derived stromal cells: significance of direct cell-to-cell contact in coculture system. Spine.

[49] Yang S.H., Wu C.C., Shih T.T., Sun Y.H., Lin F.H. (2008). *In vitro* study on interaction between human nucleus pulposus cells and mesenchymal stem cells through paracrine stimulation. Spine.

[50] Risbud M.V., Albert T.J., Guttapalli A. (2004). Differentiation of mesenchymal stem cells towards a nucleus pulposus-like phenotype *in vitro*: implications for cell-based transplantation therapy. Spine.

[51] Richardson S.M., Walker R.V., Parker S. (2006). Intervertebral disc cell-mediated mesenchymal stem cell differentiation. Stem Cells.

[52] Sakai D., Mochida J., Iwashina T. (2005). Differentiation of mesenchymal stem cells transplanted to a rabbit degenerative disc model: potential and limitations for stem cell therapy in disc regeneration. Spine.

[53] DiStefano T.J., Shmukler J.O., Danias G., Iatridis JC. (2020). The functional role of interface tissue engineering in annulus fibrosus repair: bridging mechanisms of hydrogel integration with regenerative outcomes. ACS Biomater Sci Eng.

[54] Schmitz T.C., Salzer E., Crispim J.F. (2020). Characterization of biomaterials intended for use in the nucleus pulposus of degenerated intervertebral discs. Acta Biomater.

[55] Showalter B.L., Elliott D.M., Chen W., Malhotra NR. (2015). Evaluation of an *in situ* gelable and injectable hydrogel treatment to preserve human disc mechanical function undergoing physiologic cyclic loading followed by hydrated recovery. J Biomech Eng.

[56] Kalaf E.A.G., Pendyala M., Bledsoe J.G., Sell SA. (2017). Characterization and restoration of degenerated IVD function with an injectable, *in situ* gelling alginate hydrogel: an *in vitro* and *ex vivo* study. J Mech Behav Biomed Mater.

[57] Serigano K., Sakai D., Hiyama A., Tamura F., Tanaka M., Mochida J. (2010). Effect of cell number on mesenchymal stem cell transplantation in a canine disc degeneration model. J Orthop Res.

[58] Rasoulian A., Vakiki-Tahami F., Smit TH. (2021). Linear and nonlinear biphasic mechanical properties of goat IVDs under different swelling conditions in confined compression. Ann Biomed Eng.

