# Supplementary material for: Combination of ultra-purified stem cells with an in situ-forming bioresorbable gel enhances intervertebral disc regeneration
Source: eBioMedicine. 2022 Jan 25;76:103845. doi: 10.1016/j.ebiom.2022.103845 (PMC8801983; doi:10.1016/j.ebiom.2022.103845)
Supplement: Supplementary file 1 [file mmc1.docx]

Caption for supplementary material

Supplementary Fig. 1. Histological evaluation at 4 weeks after NP tissue removal.

Supplementary Fig. 2. Measurement of disc height relative to that of the adjacent vertebra using T2-weighted, midsagittal images.

Supplementary Table. 1. Predesigned primer and probe mixes.

Supplementary Table. 2. Binucleate cell numbers per square millimeter.
