# Supplementary material for: Combination of ultra-purified stem cells with an in situ-forming bioresorbable gel enhances intervertebral disc regeneration
Source: eBioMedicine. 2022 Jan 25;76:103845. doi: 10.1016/j.ebiom.2022.103845 (PMC8801983; doi:10.1016/j.ebiom.2022.103845)
Supplement: Supplementary file 2 [file mmc2.pdf]

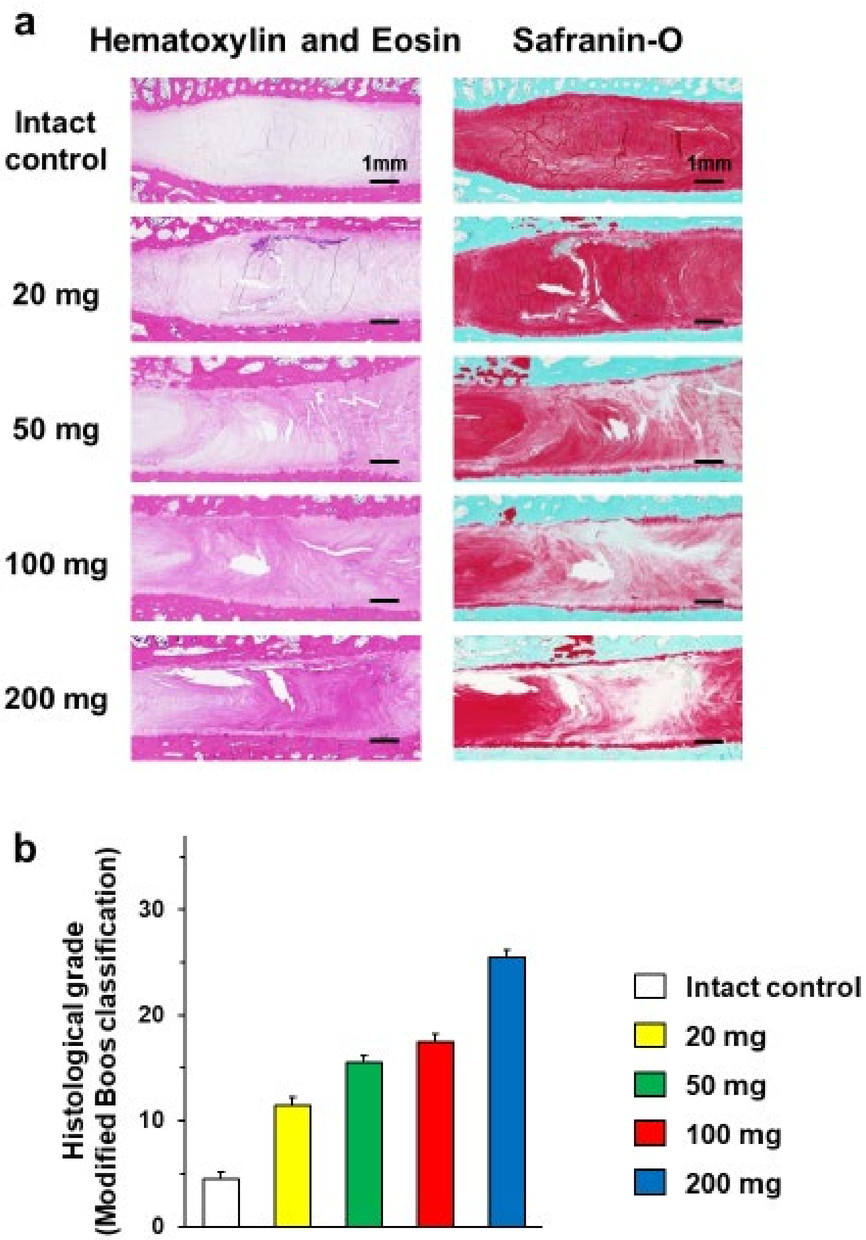


**Supplementary Fig. 1. Histological evaluation at 4 weeks after NP tissue removal. (a)**: Midsagittal sections of the nucleus pulposus (NP) tissues (intact control, 20, 50, 100, 200 mg) removed from intervertebral discs (IVDs) stained with Hematoxylin and Eosin（H&E) or safranin-O. IVD degeneration occurred with the removal of 20 mg or more of NP tissue after 4 weeks in a sheep model. Scale bar = 1 mm (all). **(b)**: Histological grades determined via modified Boos' classification. Data represent mean ± SD values.

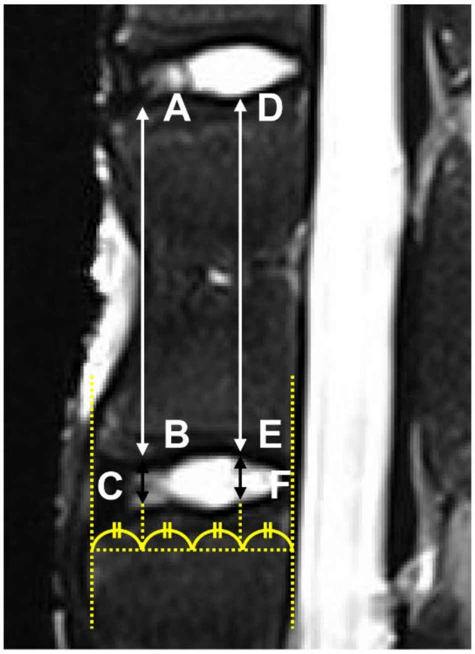


**Disc Height Index (DHI)**

$$= (BC + EF) / (AB + DE)$$

**Relative DHI**

$$= DHI_{treatment} / DHI_{Intact\ control} \times 100$$

**Supplementary Fig. 2. Measurement of disc height relative to that of the adjacent vertebra using T2-weighted, midsagittal images.** BC and EF are the anterior and posterior disc heights, respectively, and AB and DE are the adjacent vertebral body heights of cranial side. Disc height index (DHI) values were calculated as the ratio of the disc height (BC + EF) to the vertebral body height (AB + DE), and relative DHI values were calculated as the ratio of the DHI of treated intervertebral discs (IVDs) to the DHIs of intact control IVDs.

**Supplementary Table. 1. Predesigned primer and probe mixes.**

| Gene | Gene Symbol | Assay ID |
|---|---|---|
| HIF-1α | HIF1A | Hs00153153_m1 |
| GLUT-1 | SLC2A1 | Hs00892681_m1 |
| Brachyury | T | Hs00610080_m1 |
| CDMP-1 | GDF5 | Hs00167060_m1 |
| TGF-β1 | TGFB1 | Hs00998133_m1 |
| IGF-1 | IGF1 | Hs01547656_m1 |
| Type II collagen | COL2A1 | Hs00264051_m1 |
| Aggrecan | ACAN | Hs00153936_m1 |
| GAPDH | GAPDH | Hs02786624_g1 |

**Supplementary Table. 2. Binucleate cell numbers per square millimeter.**

| Intact control | REC + gel | *P*-value |
|---|---|---|
| 0·03 ± 0·07 | 0·23 ± 0·25 | 0·3311 |

Data are means ± SD. *P*-value was determined by Mann-Whitney *U* test following Welch test.
